# Multicellular Communication Networks in Myocardial Ischemia-Reperfusion Injury: Spatial and Temporal Organization of Injury and Repair

**DOI:** 10.3390/cells15201869

**Published:** 2026-10-14

**Authors:** Yiming Wang, Yuxuan Luo

**Affiliations:** Key Laboratory of Developmental Genes and Human Disease, School of Life Science and Technology, Southeast University, Nanjing 210096, China

**Keywords:** myocardial ischemia-reperfusion injury, intercellular communication, cell signaling, spatial and temporal heterogeneity, microvascular dysfunction, cardiac remodeling

## Abstract

**Highlights:**

**What are the main findings?**

• Intercellular communication after myocardial ischemia–reperfusion varies across the infarct core, border zone and remote myocardium, with the coronary microvasculature forming an interface across these regions.
• Cardiomyocytes, immune cells, vascular cells, fibroblasts, and platelets communicate through soluble mediators, extracellular vesicles, metabolic and matrix-associated cues, and direct cell–cell interactions.

**What are the implications of the main findings?**

• Defining sender–signal–recipient relationships helps distinguish causal intercellular communication from spatial association, parallel cellular responses, and cell-autonomous injury mechanisms.
• Therapeutic strategies may require phase- and compartment-specific modulation of pathological communication while preserving inflammatory resolution, vascular repair, and scar stabilization.

**Abstract:**

Myocardial ischemia–reperfusion injury (MIRI) complicates recovery after acute myocardial infarction despite timely restoration of coronary blood flow. Beyond cardiomyocyte-intrinsic oxidative stress, calcium dysregulation, mitochondrial injury, and regulated cell death, reperfusion simultaneously reorganizes communication among multiple cardiac and extracardiac cell populations. Single-cell and spatial multiomic studies further reveal marked spatial and temporal heterogeneity across the injured heart, although the causal intercellular mechanisms underlying many associations remain incompletely defined. In this Review, we examine MIRI through a multicellular communication framework and discuss interactions among cardiomyocytes, immune cells, endothelial cells, platelets, fibroblasts, vascular mural cells, and extracardiac tissues across the infarct core, border zone, coronary microvascular unit, and remote myocardium. Communication occurs through soluble danger and inflammatory signals, selected metabolites, extracellular vesicles, extracellular matrix and mechanical cues, and direct contact or junctional mechanisms. We distinguish experimentally supported intercellular pathways from spatial associations, parallel responses, and indirect regulatory mechanisms. A spatiotemporally resolved view of these networks may help identify interactions that amplify injury, impair microvascular reperfusion, or participate in resolution and repair, while guiding selective interventions that limit pathological signaling without compromising tissue recovery.

## 1. Introduction

Ischemic heart disease remains a leading cause of death and disability, and survivors of acute myocardial infarction (MI) remain at risk of heart failure and premature mortality [1,2]. Timely coronary reperfusion is essential for myocardial salvage, but restoration of epicardial flow does not invariably restore tissue perfusion. Microvascular injury after primary percutaneous coronary intervention (PCI) for ST segment elevation myocardial infarction (STEMI) is associated with adverse outcomes [3,4]. Effective strategies that reproducibly limit myocardial ischemia–reperfusion injury (MIRI) in clinical practice remain limited.

**Figure 1:**
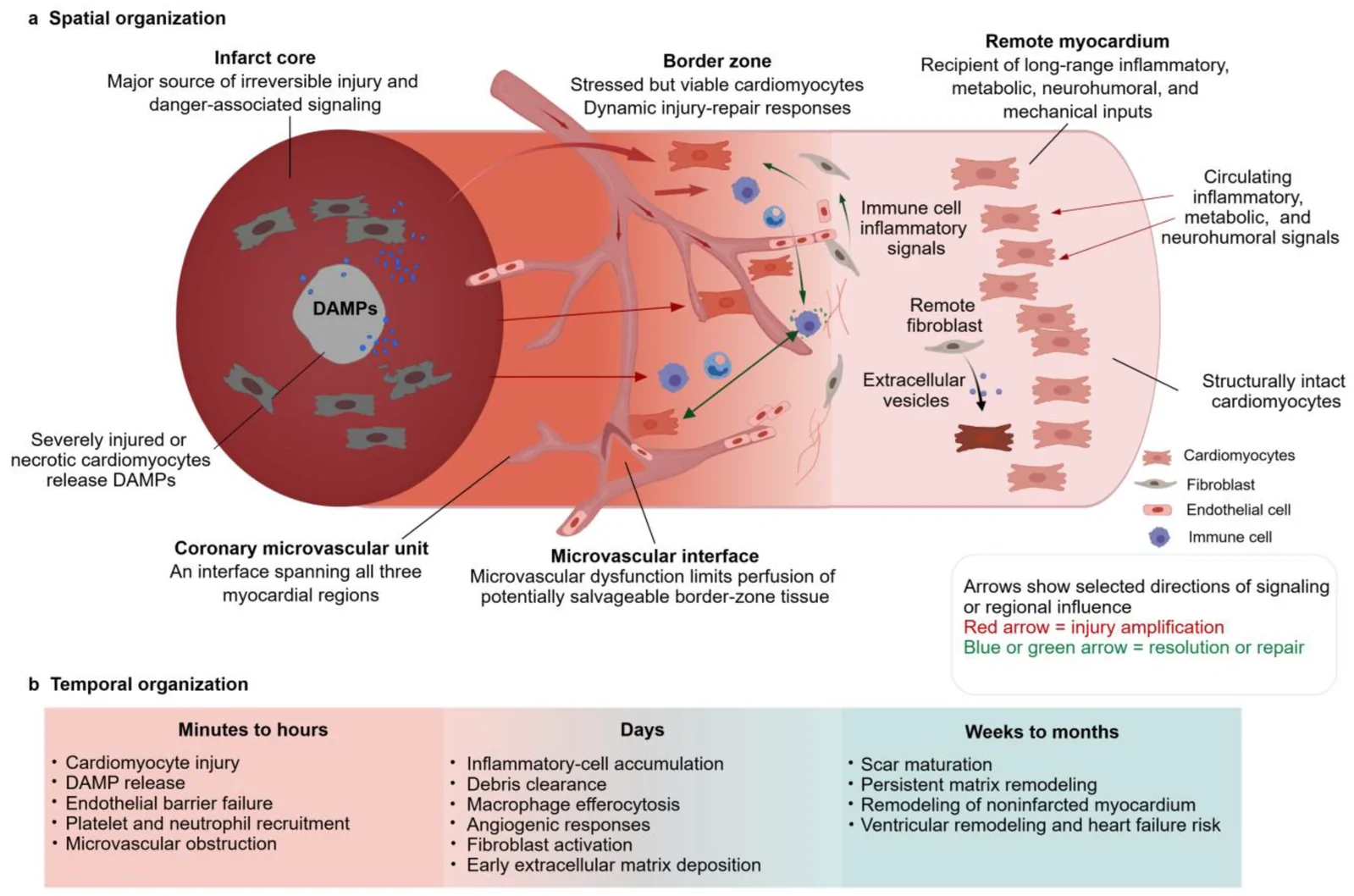
Spatial territories and temporal transitions of intercellular communication during myocardial ischemia–reperfusion injury (MIRI). (**a**) Schematic representation of the functionally coupled myocardial territories involved in MIRI. The infarct core is depicted as a source of irreversible cardiomyocyte injury and cell death, illustrated as severely injured or necrotic cardiomyocytes, with release of damage-associated molecular patterns (DAMPs). The surrounding border zone integrates injury-derived signals with local immune, endothelial, fibroblast, extracellular-matrix, metabolic, and mechanical responses. The remote myocardium receives longer-range neurohumoral, inflammatory, metabolic, and mechanical inputs, contributing to low-grade inflammation, metabolic remodeling, and chronic ventricular remodeling. The coronary microvascular unit spans the infarct core, border zone and remote myocardium and functions as an interface across these compartments. Arrows indicate representative directions of interterritorial signaling. (**b**) Temporal organization of the predominant processes during the transition from reperfusion-associated injury to repair and chronic remodeling over minutes to hours, days, and weeks to months. The spatial territories and temporal phases are dynamic, overlapping, and schematic rather than anatomically to scale. The schematic illustration was created with BioGDP.com [28].

Oxidative stress, calcium dysregulation, mitochondrial injury, regulated cell death, and inflammation contribute to MIRI [5,6]. Reperfusion also reorganizes signaling among cardiomyocytes, endothelial cells, immune cells, platelets, fibroblasts, vascular mural cells, and extracardiac tissues. Single-cell and spatial profiling reveal marked cellular and regional heterogeneity [7,8,9], but do not by themselves establish how one population influences another. Intercellular mechanisms complement established cell-autonomous injury pathways [5,10], linking intracellular disturbances to signal release, extracellular conditions, and functional responses in recipient cells. Interpreting these interactions requires distinguishing ischemic injury from reperfusion injury. Permanent coronary occlusion models sustained ischemia and subsequent inflammation and repair, whereas transient occlusion followed by reperfusion adds injury during the restoration of oxygen supply and blood flow [5,6,11]. Acute injury during early reperfusion should also be distinguished from subsequent resolution, scar formation, and remodeling. Although permanent occlusion and ischemia–reperfusion (I/R) models share inflammatory and reparative mechanisms, reperfusion alters their timing and distribution [5,6]. Reduced infarct size in an I/R model does not alone establish a reperfusion-specific effect, particularly when treatment precedes ischemia. Intervention timing, ischemia duration, area at risk, tissue perfusion, and the outcome window must therefore inform interpretation. Communication also varies across the infarct core, border zone, and remote myocardium, with the coronary microvasculature extending across all three regions (Figure 1a). Injured tissue releases damage-associated molecular patterns (DAMPs), including high-mobility group box 1 (HMGB1), ATP, and mitochondrial DNA, that influence immune, vascular, and stromal cells [11,12]. The border zone contains stressed but viable cells, while remote myocardium responds to altered loading and circulating signals [4,13,14]. Remote tissue can also act as a sender, as illustrated by fibroblast-derived exosomes promoting apoptosis in injured cardiomyocytes in experimental I/R [15].

Spatial studies characterize cellular neighborhoods in human MI [7] and, in experimental infarction, border-zone mechanosensitive responses [16,17] and cardiomyocyte-initiated type I interferon response clusters [8]. Their functional implications remain context dependent. In experimental MI, blocking von Willebrand factor (VWF) reduced monocyte infiltration but worsened cardiac function [18], cautioning against assuming that reduced immune recruitment is beneficial. Direct myocardial I/R protein profiling [9] and spatial metabolomics identifying α-galactosidase A (GLA)-dependent lipid alterations [19] further characterize regional responses to reperfusion. Tan et al. discuss how extracellular metabolic signals and immune-cell metabolic reprogramming may link early injury amplification to later resolution and repair [20]. However, molecular localization and inferred ligand–receptor interactions do not establish signal transfer or causation. Findings from permanent infarction and reperfused myocardium should therefore be distinguished, and datasets from different models and time points should not be combined into an assumed single reperfusion trajectory. These spatial differences evolve over time (Figure 1b). Early injury and vascular dysfunction overlap with immune recruitment, followed by debris clearance, vascular recovery, and scar formation [21,22,23,24,25]. Persistent inflammation or matrix remodeling can impair recovery, but immune clearance and matrix deposition are also necessary for cardiac healing [26,27]. Therapeutic interpretation therefore requires identifying the recipient-cell response and the phase in which an interaction operates.

This review focuses on intercellular mechanisms affecting acute myocardial reperfusion injury, microvascular perfusion, and the transition to resolution and repair. Later, remodeling is considered where it clarifies these mechanisms. Examples were selected for their relevance to myocardial I/R and evidence defining a sender, a transmitted signal or physical interaction, a recipient, and a functional outcome. Primary myocardial I/R studies provide the principal mechanistic evidence. Findings from permanent coronary ligation, human MI tissue, isolated hearts, and cultured cells are included where they clarify spatial organization, repair, or specific communication mechanisms, with their experimental context stated explicitly. Spatial and computational analyses are treated as hypothesis-generating unless functionally validated. This is a selective synthesis rather than an exhaustive inventory.

## 2. Intercellular Communication Networks in MIRI: Cellular Circuits Linking Injury, Repair, and Remodeling

### 2.1. Cardiomyocyte-Immune Cell Interactions: Bidirectional Signaling Between Injury Amplification and Repair

**Figure 2:**
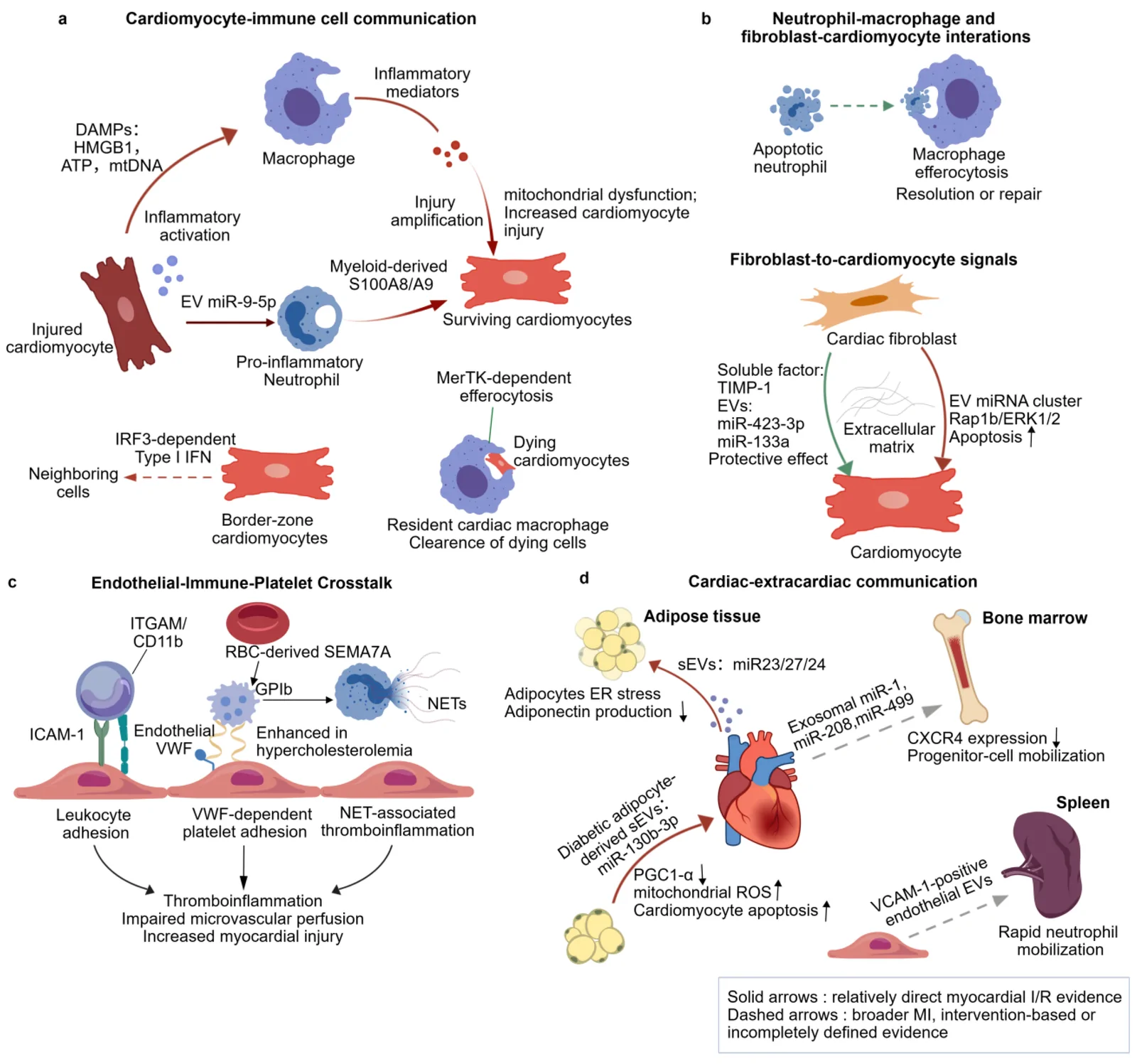
Representative local and long-range intercellular communication circuits implicated in MIRI. (**a**) Cardiomyocyte-immune cell communication. Injured cardiomyocytes release damage-associated signals, including HMGB1, ATP, and mtDNA, as well as extracellular vesicles (EVs), thereby activating neutrophils and macrophages. Immune-cell-derived mediators, including S100A8/A9, can impair mitochondrial function and viability in surviving border-zone cardiomyocytes. Conversely, IRF3-dependent type I interferon (IFN) responses initiated by border zone cardiomyocytes may influence neighboring cells. MerTK-dependent macrophage efferocytosis facilitates the removal of dying cardiomyocytes and supports tissue repair. (**b**) Neutrophil–macrophage and fibroblast–cardiomyocyte interactions. The upper circuit depicts macrophage recognition and efferocytosis of apoptotic neutrophils, a potential mechanism linking the decline of acute inflammation to resolution and repair. Direct evidence for endogenous neutrophil efferocytosis during myocardial I/R remains limited. The lower circuit summarizes fibroblast-to-cardiomyocyte communication through soluble mediators, including tissue inhibitor of metal-loproteinases-1 (TIMP-1), EV-associated miR-423-3p and miR-133a, and extracellular matrix (ECM)-mediated signaling. Depending on the cellular context and vesicular cargo, fibroblast-derived signals may be protective or injurious. The latter may involve Rap1b suppression, altered Rap1b/ERK1/2 signaling, and cardiomyocyte apoptosis. (**c**) Endothelial-immune-platelet crosstalk in the reperfused coronary microcirculation. Activated endothelial adhesion systems recruit leukocytes, and endothelial von Willebrand factor (VWF) supports platelet adhesion. Red blood cell (RBC)-derived semaphorin 7A (SEMA7A) interacts with platelet glycoprotein Ib (GPIb) and enhances platelet–neutrophil interactions. Excessive neutrophil extracellular traps (NETs) formation contributes to thromboinflammation. These overlapping processes can impair microvascular perfusion and aggravate myocardial injury, but do not constitute a single obligatory sequence. (**d**) Cardiac–extracardiac communication mediated by EVs. Cardiomyocyte-derived small extracellular vesicles (sEVs) carrying the miR-23/27/24 cluster can induce adipocyte endoplasmic-reticulum (ER) stress and reduce adiponectin production. A model-specific reverse pathway involving diabetic adipocyte-derived sEVs carrying miR-130b-3p is also shown, with increased cardiomyocyte mitochondrial ROS, reduced PGC-1α, and cardiomyocyte apoptosis. Dashed pathways represent broader myocardial infarction (MI) models, intervention-based evidence, or incompletely defined mechanisms, including myocardial EV-mediated suppression of CXCR4 in bone-marrow cells with increased progenitor-cell mobilization and VCAM-1-positive endothelial EV-mediated neutrophil mobilization from the spleen. Solid arrows indicate relatively direct evidence from myocardial I/R studies. The pathways are representative and may differ according to the donor and recipient cell states, experimental model, and disease phase. The schematic illustration was created with BioGDP.com [28].

Cardiomyocyte-immune cell interactions are a major route by which localized myocardial injury elicits inflammatory responses and subsequently engages reparative processes. These interactions begin early after reperfusion and evolve as inflammatory cell recruitment and injury amplification give way to debris clearance and tissue repair [21,23,29]. Their consequences vary with the interacting cell populations and stage of injury. Danger sensing and inflammatory injury are prominent early after reperfusion, whereas the clearance of dying cells and resolution become increasingly important as repair proceeds (Figure 2a) [21].

Cardiomyocytes initiate immune signaling when mitochondrial injury, sarcolemmal disruption, and regulated cell death release DAMPs, including mitochondrial DNA, ATP and HMGB1 [12,27]. Neutrophils, monocytes, macrophages, and resident immune cells detect these signals through pattern-recognition receptors, including Toll-like receptors, converting cardiomyocyte injury into sterile inflammation [27,30,31]. Type I interferon signaling illustrates that this communication can arise through distinct mechanisms. In MI, phagocytes can activate IRF3-dependent interferon responses after sensing necrotic cardiomyocyte-derived material (Figure 2a) [32]. More recent cell-specific genetic and spatial analyses identified border zone cardiomyocytes as major initiators of a spatially restricted type I interferon response that extends to neighboring cells [8]. Activated immune cells, in turn, affect surviving cardiomyocytes by releasing inflammatory, oxidative, proteolytic, and neutrophil extracellular trap (NET)-associated mediators [33,34,35]. Among the better defined sender–recipient mechanisms, myeloid-derived S100A8/A9 directly impairs cardiomyocyte mitochondrial function and promotes cardiomyocyte death during myocardial I/R (Figure 2a) [36]. Available evidence supports a reciprocal sequence in which cardiomyocyte injury generates extracellular danger signals that activate immune responses, while immune-cell-derived mediators can subsequently amplify injury in surviving cardiomyocytes. The spatial interferon study included coronary ligation with and without reperfusion. Its border zone findings do not establish that this circuit is specific to injury during the first minutes of reperfusion.

Cardiomyocyte-immune interactions also contribute directly to the removal of irreversibly injured cells and subsequent repair. In reperfused MI, resident MHCII-low, CCR2-negative cardiac macrophages express high levels of the phagocytic receptor MerTK and participate in the clearance of dying cardiomyocytes. Reperfusion-associated MerTK cleavage compromises this process. Macrophage MerTK deficiency reduces cardiac wound debridement and worsens infarct injury and cardiac function, whereas cleavage-resistant MerTK preserves efferocytic macrophage activity and improves repair (Figure 2a) [21]. This provides direct causal evidence that macrophage recognition and clearance of injured cardiomyocytes constitute a reparative form of cardiomyocyte-immune communication after reperfusion. Cardiomyocyte-immune crosstalk therefore includes at least two mechanistically distinct processes. Injury-associated signals released from damaged cardiomyocytes can activate and recruit immune responses, whereas immune cells can either amplify injury in viable cardiomyocytes or remove irreversibly damaged cardiomyocytes during resolution and repair. These processes should be distinguished by sender and recipient cell identity, signal direction, and disease phase rather than treated as a uniformly detrimental or reparative axis.

### 2.2. Neutrophil–Macrophage Interactions: Efferocytosis at the Inflammation–Repair Transition

Neutrophil–macrophage interactions are particularly relevant during the transition from acute inflammation to resolution. As infiltrating neutrophils undergo apoptosis, their recognition and engulfment by macrophages remove inflammatory cells and can alter macrophage programs involved in repair (Figure 2b). In a rat model involving 1 h of coronary occlusion followed by reperfusion, nanoparticles administered immediately before reperfusion-induced neutrophil apoptosis, increased macrophage uptake of apoptotic neutrophils, and improved cardiac repair [37]. Engineered neutrophil apoptotic bodies likewise target macrophages and enhance inflammation resolution [38]. These interventions establish the capacity of apoptotic neutrophil material to modify macrophage behavior, but they do not quantify the contribution of endogenous neutrophil efferocytosis during MIRI. Efficient removal of apoptotic neutrophils may therefore couple the decline of neutrophil-dominated inflammation to macrophage-mediated resolution and repair. Direct evidence defining the endogenous signals transferred from neutrophils to macrophages specifically during myocardial I/R remains limited. Future studies should distinguish the effects of neutrophil apoptosis, macrophage recognition and engulfment, and the macrophage-state changes induced by efferocytosis.

### 2.3. Endothelial-Immune-Platelet Crosstalk: A Thromboinflammatory Circuit Contributing to Microvascular Dysfunction and No-Reflow

**Figure 3:**
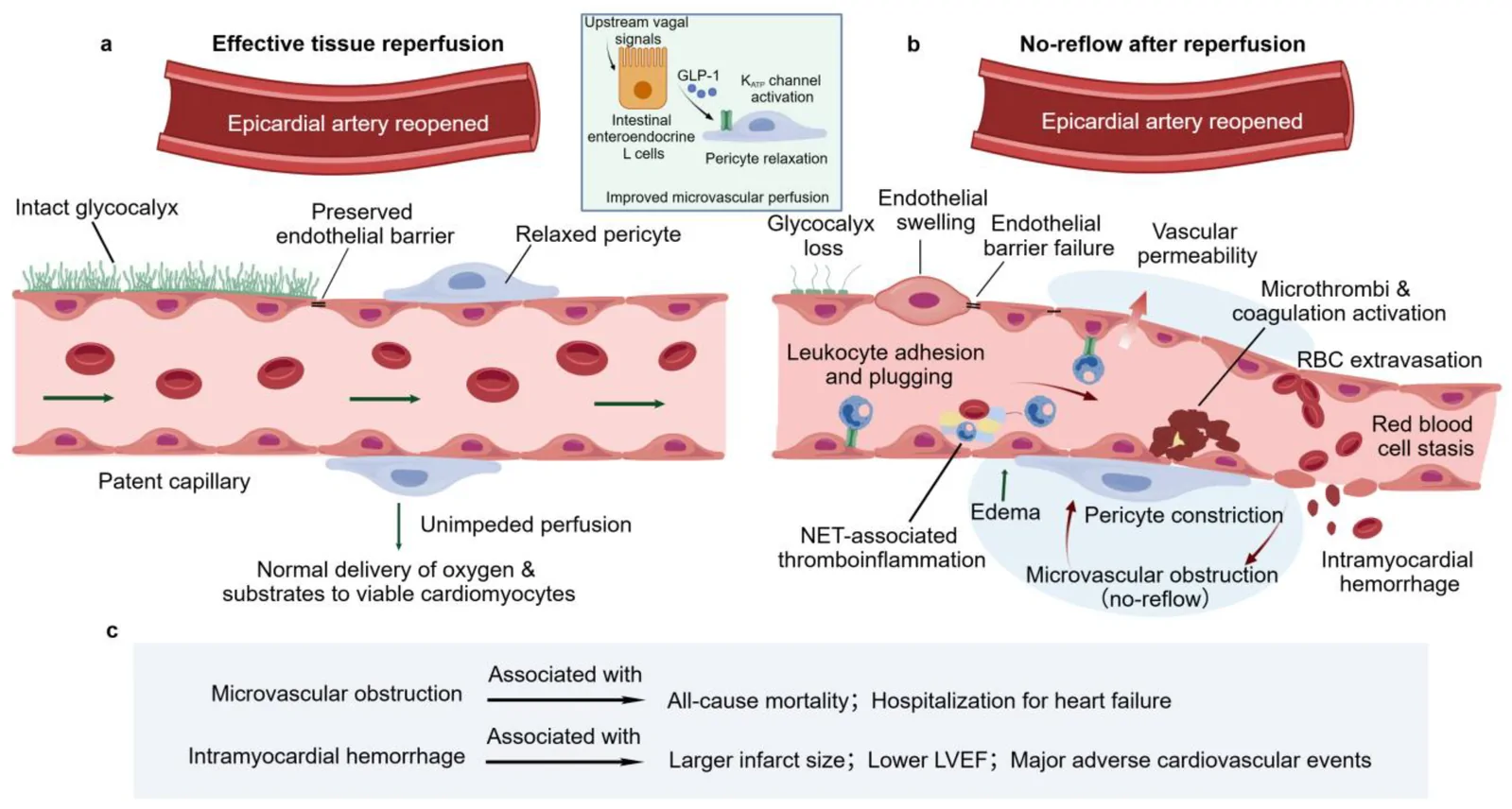
The coronary microvascular unit as a cross-compartment interface in effective tissue reperfusion and microvascular no-reflow. (**a**) Following reopening of the infarct-related epicardial artery, preservation of the endothelial glycocalyx and barrier, capillary patency, and pericyte relaxation support unimpeded microvascular flow and the delivery of oxygen and substrates to viable cardiomyocytes. The inset in panel a depicts glucagon-like peptide-1 (GLP-1) released from intestinal enteroendocrine L cells during experimental remote ischemic conditioning. Circulating GLP-1 acts on coronary pericytes, with ATP-sensitive potassium (KATP) channel activation contributing to pericyte relaxation and improved microvascular perfusion. (**b**) Despite epicardial recanalization, glycocalyx loss, endothelial barrier failure, increased vascular permeability, edema, leukocyte adhesion and plugging, neutrophil extracellular trap (NET)-associated thromboinflammation, platelet and microthrombus formation, coagulation activation, and pericyte-mediated microvascular constriction can impair capillary perfusion and cause red blood cell (RBC) stasis. Severe vessel wall disruption can also permit RBC extravasation, producing intramyocardial hemorrhage. Hemorrhage can coexist with microvascular obstruction but does not represent effective nutritive perfusion or an obligatory final step of no-reflow. (**c**) Microvascular obstruction (MVO) and intramyocardial hemorrhage (IMH) have prognostic associations with adverse remodeling and clinical outcomes. In panels (a) and (b), arrows indicate the direction of blood flow or selected mechanisms contributing to impaired perfusion. In panel (c), arrows indicate reported associations with clinical outcomes and do not imply causion. The schematic illustration was created with BioGDP.com [28].

The coronary microvascular unit extends throughout the myocardium, including the infarct core, border zone, and remote regions. After epicardial recanalization, endothelial, leukocyte, platelet, and mural-cell responses influence whether downstream capillaries deliver oxygen and substrates [4,39,40]. Glucagon-like peptide-1 (GLP-1) provides a defined extracardiac input. In experimental remote ischemic conditioning, vagal signaling evokes GLP-1 release from intestinal enteroendocrine L cells. Circulating GLP-1 acts on the coronary microvascular system, with pericyte K_ATP_ channel activation contributing to relaxation and improved perfusion (Figure 3a) [41]. Pharmacological channel blockade and pericyte-specific channel deletion support the effector role. This experimental pathway does not establish its quantitative contribution in patients.

Glycocalyx disruption reduces the protective surface layer and favors leukocyte and platelet contact with endothelium. Barrier failure permits plasma leakage and interstitial edema, which can increase extravascular compression of capillaries. Endothelial swelling, leukocyte plugging, microthrombi, and pericyte contraction further narrow the lumen and increase resistance (Figure 3b) [39,40,42]. Severe vessel wall disruption allows red blood cells to leave the vascular lumen, producing intramyocardial hemorrhage. Such extravasation requires blood to reach a damaged vessel but is not effective nutritive capillary perfusion. Hemorrhage can coexist with microvascular obstruction in a spatially heterogeneous reperfused territory [42,43]. The prognostic significance of microvascular injury differs according to its cardiac magnetic resonance (CMR)-defined phenotype. The extent of microvascular obstruction (MVO) is associated with all-cause mortality and hospitalization for heart failure, whereas an intramyocardial hemorrhage (IMH)-positive phenotype is associated with larger infarct size, lower left ventricular ejection fraction, and major adverse cardiovascular events (Figure 3c) [3,44].

Endothelial dysfunction provides a major interface for assembly of this thromboinflammatory response. Reperfusion-associated oxidative and inflammatory stress disrupts endothelial barrier integrity and increases adhesive interactions with circulating leukocytes and platelets [39,40]. Increased expression or availability of selectins, intercellular adhesion molecule 1 (ICAM-1), vascular cell adhesion molecule 1 (VCAM-1), and related adhesion systems promotes leukocyte capture, firm adhesion, and transendothelial migration [39,45]. Platelets interact with activated endothelium and leukocytes through adhesive and soluble signals, contributing to thromboinflammatory amplification. Direct cardiac I/R evidence shows that red blood cell-derived semaphorin 7A enhances platelet glycoprotein Ib (GPIb)-dependent platelet–neutrophil complex formation and myocardial thromboinflammation (Figure 2c) [46]. This mechanism identifies an additional circulating input into the platelet–neutrophil response but should not be interpreted as evidence that platelet–leukocyte aggregates alone determine reperfusion injury. Platelet–leukocyte complexes increase after myocardial I/R but are not indispensable for injury propagation [47].

NETs provide a mechanistic interface between neutrophilic inflammation, platelet activation, and coagulation. In patients with STEMI, culprit-site extracellular DNA is associated with microvascular obstruction, although this association does not establish a causal role for NETs [48]. Experimental studies demonstrate that excessive NET formation exacerbates cardiac injury, supporting a pathogenic contribution of NETs to reperfusion injury [49]. Human culprit coronary thrombi provide additional evidence for reciprocal neutrophil-platelet-coagulation signaling. NETs bearing functional tissue factor can promote thrombin generation, while thrombin-activated platelets stimulate NET formation, establishing a feed-forward thromboinflammatory interaction at the site of coronary thrombosis [50]. This study addresses culprit-artery atherothrombosis rather than downstream myocardial microvascular obstruction, and its contribution to no-reflow should not be assumed. Endothelial–platelet interactions within the reperfused microcirculation may provide a more direct route to impaired tissue perfusion. Experimental reperfused MI studies indicate that endothelial VWF-dependent platelet adhesion contributes to impaired microvascular reflow, with this response exacerbated by hypercholesterolemia (Figure 2c) [51]. The VWF findings also illustrate context dependence. Reducing platelet adhesion in reperfused microvessels [51] is not equivalent to suppressing VWF-dependent endocardial monocyte entry during infarct healing [18]. Cell type, vascular compartment, and endpoint must therefore be specified before VWF is proposed as a uniformly detrimental target. The available evidence supports microvascular dysfunction as the result of overlapping endothelial, leukocyte, platelet, and coagulation abnormalities rather than a single dominant cellular interaction. The relative contribution of each component is likely to vary with the extent of endothelial injury, inflammatory burden, thrombotic activity, and clinical context. This heterogeneity may partly explain why antiplatelet, antithrombotic, anti-inflammatory, and NET-directed approaches have not produced uniformly successful clinical strategies for preventing coronary microvascular obstruction [52].

### 2.4. Fibroblast–Cardiomyocyte Crosstalk: Paracrine and Matrix-Mediated Interactions During Repair and Remodeling

Fibroblast–cardiomyocyte crosstalk operates through soluble mediators, extracellular vesicles (EVs), and changes in the extracellular matrix (ECM), but the strength of evidence differs substantially among these mechanisms . After myocardial injury, fibroblasts undergo marked spatial and functional changes and contribute to matrix deposition and scar formation [53,54]. These changes provide the biological context for crosstalk but should not themselves be interpreted as evidence of fibroblast–cardiomyocyte communication. More direct evidence comes from fibroblast-to-cardiomyocyte paracrine signaling. In experimental I/R, the secretome of hypoxia-conditioned cardiac fibroblasts increased cardiomyocyte survival. Tissue inhibitor of metalloproteinases-1 (TIMP-1) accounted for a substantial component of this effect and engaged PI3K-AKT and ERK1/2 signaling, while administration of fibroblast secretome or TIMP-1 reduced infarct injury in vivo (Figure 2b) [55]. This study provides relatively direct evidence that fibroblast-derived soluble factors can modify cardiomyocyte susceptibility to reperfusion injury.

EVs provide a second route of fibroblast-to-cardiomyocyte signaling. During ischemic postconditioning, fibroblast-derived EVs enriched in miR-423-3p reduced cardiomyocyte apoptosis through RAP2C-dependent signaling [56]. In another experimental MI/R study, fibroblast-derived exosomal miR-133a was transferred to cardiomyocytes and suppressed ELAVL1-associated pyroptotic signaling [57]. A separate study showed that exosomes released from remote nonischemic cardiac fibroblasts aggravated reperfusion injury by delivering a functional miRNA cluster that suppressed Rap1b and altered Rap1b/ERK1/2 signaling in ischemic cardiomyocytes, promoting apoptosis (Figure 2b) [15]. These studies identify a donor cell, vesicular carrier, cargo, and cardiomyocyte response, but experimental manipulation or administration of EVs does not establish the physiological magnitude of endogenous vesicle transfer during MIRI.

Fibroblasts may also influence cardiomyocytes indirectly by modifying the ECM. Fibroblast-dependent changes in matrix composition, organization, and mechanical properties alter the extracellular environment sensed by cardiomyocyte integrins, costameres, and associated mechanotransduction systems [58]. However, evidence that specifically traces a fibroblast-derived matrix alteration to a defined cardiomyocyte response and subsequent MIRI outcome remains limited. Most detailed mechanistic studies of FAK, YAP/TAZ, talin, and stiffness-dependent signaling derive from general mechanobiology, chronic fibrosis, or post-MI remodeling rather than acute myocardial I/R. The strongest evidence for fibroblast–cardiomyocyte communication in MIRI currently comes from defined soluble and vesicular signals acting on cardiomyocytes. ECM-mediated feedback may contribute to later remodeling, but direct attribution to a specific fibroblast-to-cardiomyocyte signaling pathway in reperfused myocardium remains limited.

### 2.5. Cardiac–Extracardiac Communication Through Extracellular Vesicles

EVs provide one route by which myocardial injury can influence distant organs and systemic cell reservoirs . Direct evidence in myocardial I/R supports a heart-to-adipose signaling pathway. Cardiomyocyte-derived small extracellular vesicles (sEVs) released after MI/R carry the miR-23/27/24 cluster to adipose tissue, where they induce adipocyte ER stress and impair adiponectin production. Inhibition of EV release or cardiomyocyte-specific depletion of this miRNA cluster attenuates these remote metabolic effects (Figure 2d) [59]. Heart-to-bone marrow EV communication has also been demonstrated in experimental MI. Circulating exosomal myocardial miRNAs, particularly miR-1, miR-208, and miR-499, accumulate in bone marrow mononuclear cells, suppress C-X-C chemokine receptor 4 (CXCR4) expression, and promote progenitor-cell mobilization (Figure 2d) [60]. Endothelial EVs may also contribute to long-range immune communication after myocardial injury. Vascular cell adhesion molecule 1 (VCAM-1)-positive endothelial EVs accumulate in the spleen and promote rapid neutrophil mobilization, with loss of EV-associated VCAM-1 impairing this response (Figure 2d) [61]. These studies indicate that EVs can transmit myocardial injury-associated information beyond the heart, but the evidence is uneven across recipient organs and disease models. Claims of cardiac–extracardiac communication are strongest when donor-cell origin, in vivo trafficking, recipient-cell uptake, cargo dependence, and functional consequences are demonstrated. At present, direct evidence for such pathways in strictly defined myocardial I/R remains substantially more limited than evidence from broader MI models.

## 3. Molecular and Biophysical Mediators of Intercellular Communication in MIRI

### 3.1. DAMP-Mediated Danger Signaling: From Cellular Injury to Recipient-Cell Activation

**Figure 4:**
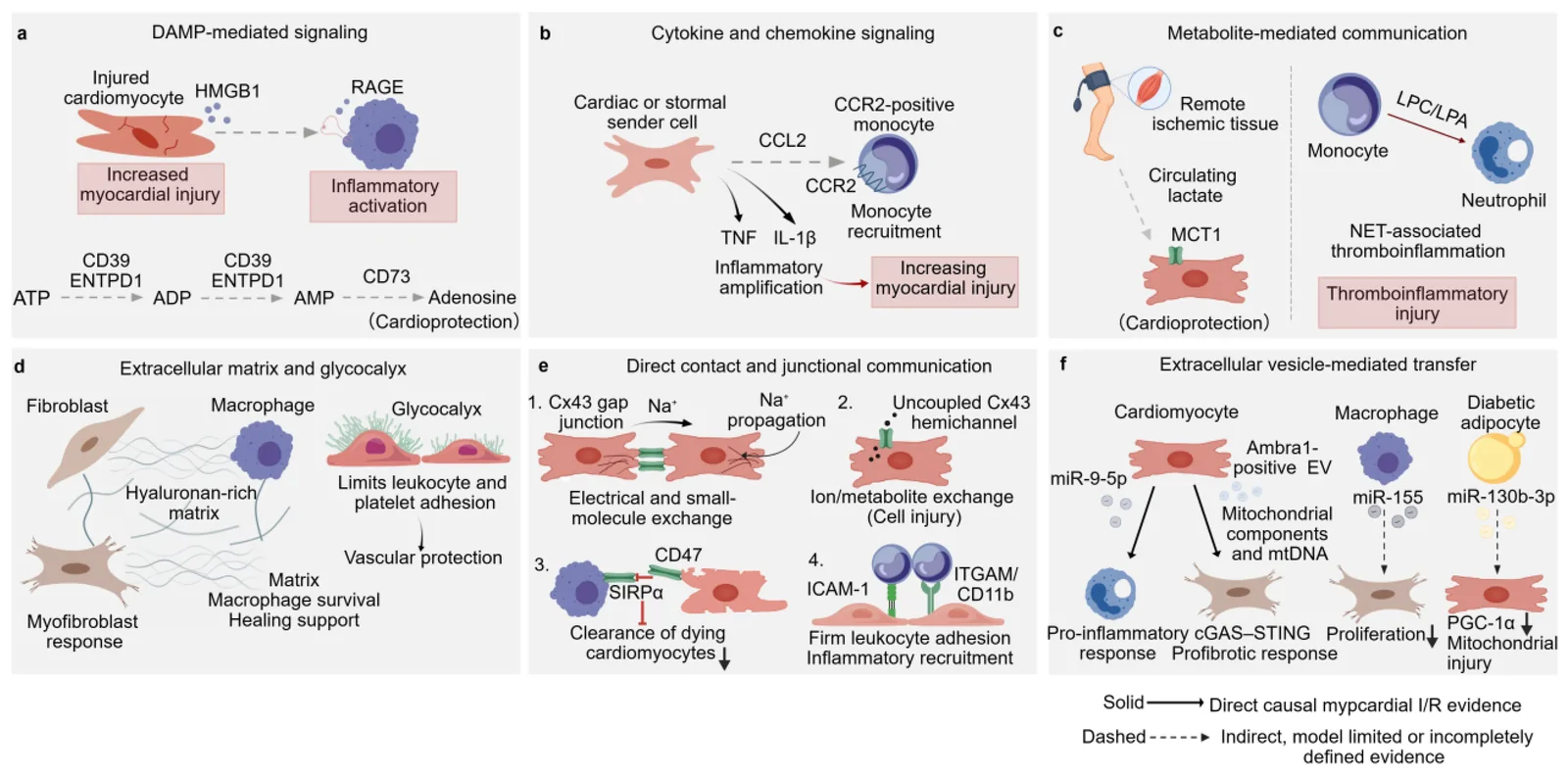
Molecular and biophysical mediators of intercellular communication in MIRI. (**a**) DAMP-mediated danger signaling. Injured cardiomyocytes release extracellular HMGB1, and HMGB1-RAGE signaling contributes to inflammatory activation and myocardial injury. The dominant recipient-cell population remains incompletely defined. Extracellular ATP and ADP are sequentially hydrolyzed by CD39/ENTPD1 to AMP and subsequently by CD73 to adenosine, thereby modifying the inflammatory, thrombotic, and cardioprotective milieu. These nucleotide pathways regulate the extracellular environment but do not necessarily define a single donor cell-to-recipient cell circuit. (**b**) Cytokine and chemokine signaling. Cardiac or stromal sender cells produce CCL2, which engages CCR2 on inflammatory monocytes and promotes monocyte recruitment. TNF and IL-1β contribute to inflammatory amplification after reperfusion. (**c**) Metabolite-mediated communication. Lactate released from remote ischemic tissue may enter cardiomyocytes through MCT1 and contribute to remote ischemic conditioning, although its precise source and quantitative contribution remain incompletely defined. In a hyperlipidemia-associated MIRI model, monocyte acyl-CoA synthetase long-chain family member 1 (ACSL1)-dependent metabolic reprogramming increases LPC/LPA production, which activates neutrophils and promotes NET-associated thromboinflammation. (**d**) Extracellular-matrix and glycocalyx-mediated regulation. Hyaluronan-rich extracellular matrix influences macrophage survival and the myofibroblast response after reperfusion. The endothelial glycocalyx acts as a physical and biochemical barrier that limits leukocyte and platelet adhesion. (**e**) Direct-contact and junctional communication. Cx43-containing gap junctions permit electrical and small-molecule exchange between adjacent cardiomyocytes and may facilitate the propagation of ionic imbalance and reperfusion-associated injury. Unopposed Cx43 hemichannels alter the extracellular ionic and metabolic environment but do not directly connect two adjacent cells. Endothelial ICAM-1/VCAM-1 and leukocyte ITGAM/CD11b mediate firm leukocyte adhesion. Cardiomyocyte CD47 engages macrophage SIRPα to restrain phagocytic clearance. (**f**) Extracellular vesicle-mediated transfer. Cardiomyocyte-derived EVs carrying miR-9-5p are taken up by neutrophils and aggravate early reperfusion injury. Ambra1-positive cardiomyocyte-derived EVs containing mitochondrial components, including mtDNA, are internalized by cardiac fibroblasts and activate cGAS-STING-dependent profibrotic responses during postischemic remodeling. Macrophage-derived miR-155-containing exosomes suppress fibroblast proliferation in a permanent coronary ligation MI model. In the context of diabetes or metabolic dysfunction, adipocyte-derived sEVs enriched in miR-130b-3p increase cardiomyocyte mitochondrial ROS and apoptosis, in part through suppression of PGC-1α. The schematic illustration was created with BioGDP.com [28].

DAMPs constitute an upstream route by which focal cellular injury elicits responses in neighboring and circulating cells during MIRI . Within minutes to hours after reperfusion, necrotic or severely stressed cardiomyocytes and other injured cardiac cells release intracellular molecules into the extracellular space. Ligand–receptor interactions mediate recipient-cell sensing. These include pattern recognition receptors for selected DAMPs, the HMGB1 receptor RAGE, and purinergic receptors for extracellular nucleotides. DAMP-mediated communication requires evidence of extracellular release, recognition by a distinct recipient cell population, and functional consequences beyond intracellular stress responses in the injured cell itself. HMGB1 is among the best-characterized extracellular DAMPs in myocardial I/R. Experimental inhibition of HMGB1 reduces injury, and direct evidence implicates signaling through the receptor for advanced glycation end products (RAGE) in inflammatory activation and myocardial injury (Figure 4a) [62,63]. Broader evidence also implicates Toll-like receptors and additional recipient-cell populations in HMGB1 signaling [62], but these interactions should be assigned only where cell-specific evidence is available.

Extracellular ATP and ADP represent another class of injury-associated signals. Acute membrane disruption and cellular stress can increase extracellular nucleotide availability, allowing purinergic receptors on immune cells, platelets, endothelial cells, and cardiomyocytes to modify inflammatory, thrombotic, and cellular responses [64,65]. Extracellular nucleotide concentrations are further regulated by CD39/ENTPD1, which hydrolyzes ATP and ADP to AMP, and CD73, which converts AMP to adenosine. Genetic and pharmacological studies in cardiac I/R and ischemic preconditioning demonstrate that CD39- and CD73-dependent nucleotide metabolism can limit infarct injury and contribute to adenosine-mediated cardioprotection (Figure 4a) [66,67]. These studies establish extracellular nucleotide metabolism as an important modifier of the reperfused myocardial environment, although they do not define a single donor cell-to-recipient cell circuit.

Mitochondrial components can function as intercellular danger signals when they leave the injured cardiomyocyte and reach a defined recipient cell. The strongest direct evidence in myocardial I/R comes from cardiomyocyte-derived Ambra1-positive small extracellular vesicles (sEVs). These vesicles contain mitochondrial components, including mtDNA, are internalized by cardiac fibroblasts, and activate fibroblast cyclic GMP-AMP synthase (cGAS)-stimulator of interferon genes (STINGs) signaling and profibrotic responses (Figure 4f) [68]. Cardiac-specific reduction of Ambra1 reduced the release of mitochondria-containing EVs and their uptake by fibroblasts, providing causal evidence for cardiomyocyte-to-fibroblast transfer of mitochondrial components after myocardial I/R [68].

Inflammasome activation represents a downstream recipient-cell response to sterile danger rather than a DAMP. Cardiac fibroblast inflammasome activation has been implicated in MIRI [69], indicating that resident stromal cells can respond to injury-associated extracellular cues. However, unless the upstream DAMP, its extracellular release, and receptor-dependent sensing are defined, inflammasome activation alone should not be interpreted as a complete intercellular communication pathway. More broadly, DAMP-mediated communication in MIRI is best supported when an injury-derived extracellular signal can be traced from its cellular source to a defined recipient response. HMGB1 provides direct evidence for extracellular danger signaling during cardiac I/R, extracellular nucleotide metabolism modifies the inflammatory and cardioprotective milieu, and vesicular transfer of mitochondrial components provides a defined cardiomyocyte-to-fibroblast pathway. Intracellular cGAS-STING activation, mitophagy, ferroptosis, and inflammasome activation remain relevant when they regulate signal production or constitute a defined recipient-cell response. They should not themselves be classified as intercellular communication.

### 3.2. Cytokine and Chemokine Circuits: Leukocyte Recruitment and Inflammatory Amplification

DAMP sensing is followed by cytokine and chemokine production that regulates leukocyte recruitment, inflammatory amplification, and subsequent tissue responses. Interpretation of these pathways requires identification of the cellular source, receptor-bearing recipient, and functional consequence. Concurrent cytokine expression or pathway activation alone does not establish a defined intercellular circuit [27]. The CCL2-CCR2 axis provides one of the clearest mechanisms for recruiting inflammatory monocytes after myocardial injury. In patients with STEMI, CCR2-targeted imaging demonstrates increased CCR2-targeted tracer uptake within injured myocardium, consistent with accumulation of CCR2-positive monocytes and macrophages, although it does not identify the cellular source of CCL2 (Figure 4b) [70]. Experimental myocardial I/R studies further implicate CCL2-CCR2 signaling in inflammatory cell accumulation and subsequent macrophage and fibrotic responses [71]. Following myocardial injury, CCR2+ resident cardiac macrophages promote monocyte recruitment through myeloid differentiation primary response 88 (MYD88)-dependent programs, whereas CCR2− resident macrophages limit this process and influence monocyte fate and cardiac outcomes [72].

TNF and IL-1β contribute to inflammatory amplification after reperfusion. After I/R, their spatial distribution changes over time, with early vascular-associated expression followed by more prominent inflammatory cell localization during healing [73]. Genetic loss of TNF reduces NF-κB activation, chemokine and adhesion-molecule expression, leukocyte infiltration, and acute MIRI, supporting a functional role for TNF in early inflammatory amplification (Figure 4b) [74]. Myeloid Dectin-1 signaling likewise increases CXCL1/granulocyte colony-stimulating factor (G-CSF)-associated neutrophil recruitment and aggravates experimental MIRI [29]. IL-1 signaling can also involve resident stromal cells. Cardiac fibroblast inflammasome activation contributes causally to MIRI and IL-1β-associated inflammatory responses [69,75]. However, inflammasome activation alone does not define the upstream sender–recipient pathway, and the dominant source and recipient populations for IL-1β are likely to vary with time and tissue compartment. IL-6 signaling illustrates why cytokine activity should not be treated as uniformly detrimental. In a rat model of reperfused MI, selective inhibition of IL-6 trans-signaling with soluble gp130-Fc (sgp130Fc) reduced neutrophil and macrophage infiltration, infarct size, and later cardiac dysfunction, whereas pan-IL-6 antagonism did not provide the same benefit [76]. These findings support functional differences between IL-6 trans-signaling and classic signaling, but they do not identify the dominant IL-6-producing and responding cell populations in MIRI. Evidence for cytokine and chemokine signaling in MIRI is strongest when ligand–receptor activity can be linked to a defined inflammatory function, as in CCR2-dependent monocyte recruitment and TNF or IL-1-associated inflammatory amplification. Spatial and single-cell datasets can identify candidate interactions, but causal assignment requires cell-source-specific perturbation, receptor dependence, and functional validation during reperfusion.

### 3.3. Metabolites and Redox Cues: Distinguishing Intercellular Signals from Cell-Autonomous Metabolic Injury

I/R profoundly alters myocardial metabolism and redox state, but most metabolic changes do not constitute intercellular communication. A metabolite should be considered an intercellular signal only when its extracellular availability or transfer from a defined source can be linked to uptake or sensing by a recipient cell and a functional response. Intracellular metabolic flux, mitochondrial ROS production, and protein acylation or lactylation within the same cell should be considered cell-autonomous unless such a transfer step is demonstrated.

Lactate illustrates this distinction. In myocardial I/R, monocarboxylate transporter 4 (MCT4) inhibition at reperfusion alters cardiomyocyte pyruvate-lactate handling, increases mitochondrial pyruvate oxidation, and improves myocardial salvage [77]. This is primarily a cell-autonomous metabolic mechanism rather than evidence of lactate-mediated intercellular communication. By contrast, remote ischemic conditioning increases circulating and myocardial lactate, and inhibition of monocarboxylate transporter 1 (MCT1)-dependent myocardial lactate uptake attenuates cardioprotection, supporting lactate as a candidate circulating signal between remote ischemic tissue and the heart (Figure 4c) [78]. The precise tissue source of the circulating lactate and its quantitative contribution relative to other humoral mediators, however, remain incompletely defined.

Immune-cell metabolism may generate extracellular metabolites capable of acting on other cardiac or inflammatory cells, although direct evidence remains limited. Pharmacological induction of macrophage itaconate production has been reported to reduce MIRI, and conditioned-medium experiments support the capacity of macrophage-derived metabolites to modify cardiomyocyte survival [79]. However, because this pathway was elicited pharmacologically and the conditioned medium contains multiple potential mediators, the physiological magnitude and specificity of endogenous macrophage-to-cardiomyocyte itaconate transfer remain uncertain.

More direct evidence for metabolite-mediated cellular communication comes from lipid metabolites in hyperlipidemia-associated myocardial I/R. Monocytic acyl-CoA synthetase long-chain family member 1 (ACSL1)-dependent metabolic reprogramming increases lysophosphatidylcholine/lysophosphatidic acid (LPC/LPA) production, and monocyte-derived LPC/LPA promotes neutrophil activation and NET-associated thromboinflammation (Figure 4c) [80]. This study provides a defined metabolic source and recipient-cell response, although the mechanism was demonstrated in a hyperlipidemic context and should not be generalized to all forms of MIRI. Metabolite-mediated intercellular communication in MIRI is supported by fewer mechanisms than the broader literature on metabolic remodeling might suggest. Convincing evidence requires a defined extracellular metabolite source, a route of delivery or uptake, a recipient-cell response, and functional interruption of that pathway. Metabolic changes confined to a single cell remain important determinants of MIRI but should not be classified as intercellular communication without evidence of extracellular transfer or recipient-cell sensing.

### 3.4. Extracellular Matrix, Glycocalyx, and Mechanical Cues as Extracellular Contexts for Intercellular Communication

The ECM provides a structural and biochemical environment in which soluble mediators, mechanical forces, and spatial constraints are presented to cells within injured myocardium. ECM components can bind cytokines, chemokines, and growth factors and influence their local availability, while changes in matrix composition and mechanics alter how immune, stromal, vascular, and myocardial cells respond to their surroundings [58,81]. These functions can modify intercellular communication, but matrix remodeling itself should not be considered a cell–cell signaling mechanism unless matrix changes generated by one cell population are sensed by another cell type and induce a functional response.

Direct evidence for matrix-dependent multicellular effects in myocardial I/R is supported by studies of hyaluronan. Hyaluronan synthase 2 (Has2)-dependent hyaluronan synthesis after cardiac I/R supports macrophage survival and the myofibroblast response, whereas genetic or pharmacological reduction in hyaluronan impairs healing (Figure 4d) [82]. In vitro evidence further indicates reciprocal interactions between macrophages and fibroblasts within this hyaluronan-dependent environment. These findings support a functional role for the ECM in immune–stromal interactions after reperfusion, although they do not define a simple unidirectional sender–signal–recipient pathway. Mechanical cues may also transmit information between cell populations when one population alters matrix stiffness, tension, or deformation and another senses the resulting mechanical change. However, direct causal evidence for such endogenous cell-to-cell mechanical communication during myocardial I/R remains limited.

The endothelial glycocalyx represents a distinct extracellular interface between the vascular wall and circulating cells. Experimental cardiac I/R causes shedding of glycocalyx components, including syndecan-1 and heparan sulfate. In isolated perfused guinea pig hearts subjected to global I/R, sevoflurane-mediated preservation of the glycocalyx attenuated postischemic vascular permeability and reduced leukocyte and platelet adhesion (Figure 4d) [83,84]. These findings support the glycocalyx as a physical and biochemical restraint on blood cell–endothelial interactions rather than as a conventional soluble signaling pathway.

Overall, the ECM and glycocalyx influence intercellular communication by controlling signal presentation, mechanical context, cellular positioning, and physical access between interacting cells. In MIRI, the strongest evidence currently concerns hyaluronan-dependent immune–stromal responses and glycocalyx-dependent blood cell–endothelial interactions. More elaborate ECM-mediated mechanical circuits remain biologically plausible but require cell-specific perturbation of matrix production or mechanics together with defined recipient-cell responses in reperfusion models.

### 3.5. Direct Contact and Junctional Communication in MIRI

Direct physical interactions provide a distinct mode of intercellular communication during MIRI. The strongest evidence involves connexin-based coupling between adjacent cardiomyocytes and adhesion- or receptor-dependent interactions among inflammatory, vascular, and myocardial cells. Cx43-containing gap junctions provide direct cytoplasmic continuity between adjacent cardiomyocytes and permit electrical and small-molecule exchange. During I/R, changes in Cx43 localization, phosphorylation, and channel function alter both electrical coupling and the propagation of injury between neighboring cardiomyocytes. Experimental studies indicate that gap-junctional communication can facilitate the spread of reperfusion-associated hypercontracture and cell injury, including transfer of Na^+^ from injured to adjacent cardiomyocytes (Figure 4e). Pharmacological disruption of gap-junctional communication during early reperfusion can limit this propagation under experimental conditions [85]. Unopposed Cx43 hemichannels can open during ischemic stress and permit ion and metabolite exchange between the cardiomyocyte cytoplasm and extracellular space. Selective inhibition of Cx43 hemichannels with Gap19 reduces ionic imbalance and cell injury in experimental I/R models (Figure 4e) [86]. Hemichannel activity is therefore relevant to intercellular signaling by altering the extracellular milieu, but it should not be classified as direct cell–cell communication because the channel does not physically connect two adjacent cells.

Direct adhesion also regulates communication between circulating inflammatory cells and the reperfused myocardium. Adhesion molecules including endothelial ICAM-1 and VCAM-1 and leukocyte integrins mediate firm leukocyte-endothelial contact and transendothelial migration during myocardial I/R (Figure 4e) [45]. Recent evidence identified ITGAM/CD11b-dependent immune–endothelial interactions as a contributor to reperfusion injury, as disruption of this pathway reduced inflammatory-cell infiltration, infarct size, and cardiac dysfunction after myocardial I/R [87]. These interactions are not merely structural. Disruption of specific adhesion pathways alters neutrophil recruitment and myocardial injury, although redundancy among adhesion systems limits attribution to a single ligand–receptor pair.

Contact-dependent receptor–ligand signaling can also regulate the fate of injured cardiomyocytes. After myocardial I/R, increased cardiomyocyte CD47 engages the inhibitory phagocytic receptor signal regulatory protein α (SIRPα) on macrophages and restrains clearance of dying cardiomyocytes. Acute blockade of CD47 increases macrophage phagocytosis of injured cardiomyocytes, accelerates inflammatory resolution, and improves infarct and functional outcomes (Figure 4e) [88]. This pathway provides direct evidence for a juxtacrine cardiomyocyte–macrophage interaction in reperfused myocardium. Evidence that tunneling nanotubes mediate transfer of intact organelles during endogenous MIRI remains limited. Such a mechanism should not be inferred without in vivo tracing and pathway-specific disruption. Direct-contact communication in MIRI therefore encompasses mechanistically distinct processes. Gap junctions permit direct electrical and molecular exchange between adjacent cardiomyocytes, adhesion systems regulate physical interactions between vascular and inflammatory cells, and membrane-bound receptor–ligand pairs can determine recipient-cell behavior during clearance and repair. Connexin hemichannels influence the extracellular signaling environment but are mechanistically distinct from direct cell–cell coupling.

### 3.6. Extracellular Vesicles as Intercellular and Long-Range Signal Carriers

EVs provide a membrane-protected route for transferring miRNAs, proteins, lipids, and mitochondrial components between cells and across tissue compartments. Cardiomyocyte-derived EVs carrying miR-9-5p are taken up by infiltrating neutrophils and promote a pro-inflammatory neutrophil phenotype through SOCS5/SIRT1-dependent JAK2/STAT3 and NF-κB signaling, aggravating early reperfusion injury (Figure 4f) [89]. The combination of donor-cell identification, cargo manipulation, recipient-cell uptake, and functional testing provides relatively direct evidence for cardiomyocyte-to-neutrophil EV communication during MIRI. However, the association between circulating EV miR-9-5p and clinical outcomes after STEMI does not establish that this pathway makes a comparable contribution in patients.

These examples represent selected EV-mediated circuits relevant to cardiac injury and reperfusion rather than an exhaustive representation of the broader cardiovascular EV communication network [90]. An additional macrophage-to-fibroblast pathway involves EV-associated miR-155. In a permanent coronary ligation MI model, macrophage miR-155-containing exosomes suppressed fibroblast proliferation and increased inflammatory responses (Figure 4f) [91]. This pathway is relevant to infarct healing but does not establish an acute reperfusion-specific effect.

In the setting of metabolic disease, adipocyte-derived EVs may provide a long-range extracardiac-to-cardiac pathway. Diabetic adipocyte-derived sEVs enriched in miR-130b-3p increased cardiomyocyte mitochondrial ROS production and apoptosis by suppressing PGC-1α, exacerbating MIRI (Figure 4f) [92]. An earlier study showed that transplantation of diabetic adipose tissue into nondiabetic mice increased susceptibility to MIRI. Systemic or intramyocardial administration of sEVs derived from diabetic serum or dysfunctional adipocytes reproduced this phenotype, whereas pharmacological inhibition of sEV biogenesis attenuated MIRI in diabetic mice [93]. This mechanism should be interpreted specifically in the context of diabetes or metabolic dysfunction rather than generalized to all forms of MIRI. Overall, EV-mediated communication in MIRI is best supported when donor-cell origin, in vivo transfer, recipient-cell uptake, cargo dependence, and functional consequences are demonstrated under endogenous or minimally manipulated conditions. Exogenous or engineered EV administration can establish delivery potential and therapeutic plausibility but should not be interpreted as evidence that the corresponding endogenous pathway is quantitatively important during reperfusion.

## 4. Translational Implications: From Single-Pathway Cardioprotection to Spatiotemporal Network Modulation

### 4.1. Why Cardioprotective Strategies Have Shown Limited Clinical Translation

**Figure 5:**
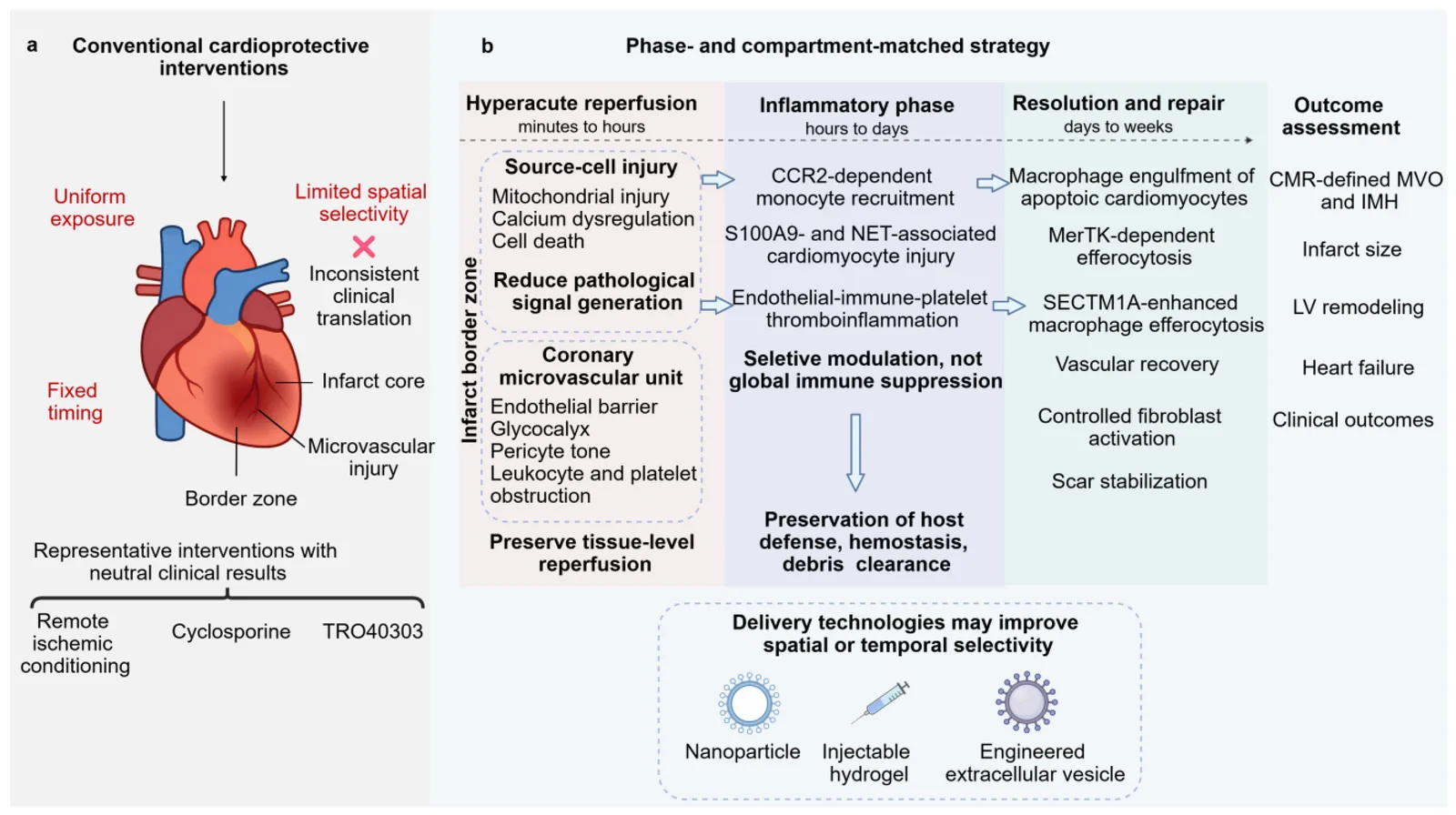
From conventional cardioprotective strategies to phase- and compartment-matched modulation of myocardial ischemia–reperfusion injury (MIRI). (**a**) Conventional cardioprotective interventions may have limited spatial selectivity and inconsistent clinical translation. These approaches include pharmacological interventions and remote ischemic conditioning. The infarct core, border zone, and coronary microvascular unit differ in cellular viability, injury mechanisms, and reparative potential. Representative interventions with neutral clinical results include remote ischemic conditioning, cyclosporine, and TRO40303. (**b**) Conceptual framework for a phase- and compartment-matched therapeutic strategy. During hyperacute reperfusion, source-cell injury, including mitochondrial injury, calcium dysregulation, and cell death, may be targeted to reduce pathological signal generation and preserve tissue-level reperfusion across the infarct border zone and coronary microvascular unit. During the inflammatory phase, selective modulation of CCR2-dependent monocyte recruitment, S100A9- and NET-associated cardiomyocyte injury, and endothelial-immune-platelet thromboinflammation should be considered while preserving host defense, hemostasis, and debris clearance. During resolution and repair, appropriately timed interventions may promote macrophage engulfment of apoptotic cardiomyocytes, MerTK-dependent and SECTM1A-enhanced efferocytosis, vascular recovery, controlled fibroblast activation, and scar stabilization. Nanoparticles, injectable hydrogels, and engineered extracellular vesicles may improve spatial or temporal selectivity, but these delivery platforms should be distinguished from the therapeutic mechanisms themselves. Outcome assessment may include CMR-defined microvascular obstruction and intramyocardial hemorrhage, infarct size, left ventricular (LV) remodeling, heart failure, and other clinical outcomes. The framework is conceptual and does not represent a validated treatment algorithm. Arrows indicate proposed transitions between phases and conceptual relationships among selected elements rather than an obligatory clinical sequence. The schematic illustration was created with BioGDP.com [28].

Despite decades of experimental investigation, cardioprotective strategies targeting oxidative stress, inflammation, ischemic conditioning, or mitochondrial injury have shown limited and inconsistent clinical benefit [6,10,94]. Several representative trials in STEMI patients undergoing primary PCI illustrate this limitation. Remote ischemic conditioning failed to reduce cardiac death or hospitalization for heart failure at 12 months in CONDI-2/ERIC-PPCI [95]. Cyclosporine-mediated inhibition of mitochondrial permeability transition pore (mPTP) opening did not improve clinical outcomes or prevent adverse left ventricular remodeling in CIRCUS [96]. The mitochondria-targeted compound TRO40303 did not reduce enzymatic or CMR-defined infarct size in MITOCARE (Figure 5a) [97]. Remote ischemic conditioning involves multiple pathways, so its neutral result should not be interpreted as a failure of single target treatment. Several features of MIRI may contribute to this translational gap. The process is rapid, spatially heterogeneous, and multicellular, so treatment efficacy depends on when an intervention is delivered, which tissue compartment it reaches, and whether it modifies injury-amplifying or repair-associated signaling.

Several key events, including oxidative stress, calcium overload, mPTP opening, and early endothelial barrier dysfunction, occur within the first minutes of reperfusion [98,99,100,101]. By the time many interventions are administered clinically, critical injury-amplifying processes may already be established. Conversely, sustained suppression of inflammation or stress-responsive pathways may interfere with debris clearance, vascular repair, and scar stabilization. In addition, the infarct core, border zone, coronary microvascular unit, and remote myocardium differ substantially in cellular viability, metabolic state, inflammatory activity, and reparative potential [7,102]. A uniformly delivered treatment may therefore fail to reach the relevant compartment or may inhibit beneficial responses in adjacent tissue.

Therapeutic response may also be limited when an intervention attenuates one injury process but leaves parallel mechanisms active or disrupts compensatory repair. These challenges are further complicated by the limited clinical relevance of standardized experimental models, as patients differ in age, metabolic status, ischemic duration, prior treatment exposure, and reperfusion timing, all of which can modify injury evolution and treatment response. The central challenge is not simply to identify a pathogenic pathway, but to determine which communication circuit should be modulated, in which myocardial compartment, and at what stage of injury and repair.

### 4.2. Spatially and Temporally Actionable Interfaces: The Border Zone and Coronary Microvascular Unit

The infarct border zone may offer a therapeutic interface during reperfusion and early repair. Unlike the infarct core, where cardiomyocyte death is often irreversible, the border zone contains stressed but potentially salvageable myocardium exposed to dynamic metabolic, inflammatory, vascular, and mechanical perturbations [8,16,103]. Its therapeutic relevance could therefore extend beyond acute cardiomyocyte preservation. In this region, immune-cell recruitment, endothelial dysfunction, fibroblast activation, ECM remodeling, and residual cardiomyocyte viability converge, making it a plausible site at which injury propagation may still be redirected toward repair.

The coronary microvascular unit may provide a complementary cross-compartment therapeutic interface. Restoration of epicardial coronary flow does not guarantee effective capillary perfusion because endothelial barrier failure, glycocalyx disruption, leukocyte plugging, platelet–neutrophil interactions, NET-associated thrombosis, complement-coagulation activation, and pericyte constriction can sustain no-reflow despite arterial recanalization [4,42,104,105]. Clinical CMR and angiographic studies support the prognostic importance of microvascular obstruction after STEMI [106], but prior attempts to treat no-reflow or microvascular obstruction have produced inconsistent results (Figure 5a) [4,107]. Myocardial salvage may therefore require protection of viable border zone myocardium together with restoration of microvascular patency rather than treatment directed exclusively at cardiomyocyte death.

These considerations should not be interpreted as evidence that the border zone or microvascular unit is already a validated therapeutic target in all patients. Instead, they provide a spatial rationale for future interventions matched to the compartment in which injury remains biologically modifiable. Such interventions must also be timed to the window during which viable myocardium can still be salvaged or evolving microvascular obstruction can still be reversed. Molecular imaging, cardiac magnetic resonance assessment of microvascular obstruction, and region-responsive delivery technologies may help identify patients in whom such approaches are most likely to be effective.

### 4.3. Targeting Intercellular Circuits Across Different Phases of MIRI

Therapeutic development should distinguish cell-autonomous injury mechanisms from intercellular signaling networks and combine interventions only when they target complementary mechanisms within clearly defined therapeutic windows (Figure 5b). Preclinical myocardial I/R studies provide proof of principle for this approach. Cyclophilin D (CypD)-dependent mitochondrial injury is most prominent early after reperfusion, whereas CCR2-dependent inflammatory injury becomes more prominent later (Figure 5b). Simultaneous nanoparticle-based targeting of these two processes produced greater infarct limitation than either intervention alone [108]. This finding supports the potential value of combining source-cell protection with modulation of downstream inflammatory responses, but it does not establish that broad multitarget therapy will necessarily improve clinical outcomes.

During the inflammatory phase, therapeutic targets should be selected from causally defined cellular interactions rather than broad suppression of immune activity. Neutrophil S100A9 lactylation provides one example. Genetic and pharmacological evidence indicates that this modification increases neutrophil migration and cardiac recruitment after myocardial I/R. S100A9 released during NET formation can also impair cardiomyocyte mitochondrial function and promote cell death (Figure 5b) [109]. Microvascular thromboinflammatory pathways involving endothelial dysfunction, leukocytes, platelets, and NETs may provide additional targets, but their overlapping functions in host defense, hemostasis, and tissue repair argue against indiscriminate inhibition. As inflammation subsides, therapeutic priorities may shift toward effective clearance of dying cells and restoration of tissue repair. In myocardial I/R, macrophage-enriched SECTM1A promotes macrophage efferocytosis of apoptotic cardiomyocytes, and recombinant SECTM1A enhances efferocytosis and improves cardiac recovery (Figure 5b) [110]. This provides a more direct example of a phase-relevant reparative intervention than nonspecific stimulation or suppression of macrophage activity. However, the appropriate timing and duration of such interventions remain critical because inflammatory clearance, vascular repair, scar formation, and matrix remodeling are interdependent processes.

Delivery technologies may improve the spatial and temporal selectivity of these interventions, but they should be distinguished from therapeutic mechanisms themselves (Figure 5b). For example, local hydrogel delivery of CD39 and CD73 reduced innate immune activation and preserved cardiac function in experimental myocardial I/R [111], whereas injectable hydrogels carrying targeted, miR-222-engineered EVs improved experimental I/R injury and remodeling [112]. These studies demonstrate preclinical delivery feasibility rather than validation of a general communication-network treatment strategy. Accordingly, the translational priority is to identify either a causal cellular interaction or a defined point at which cell-autonomous injury generates a pathological intercellular response. Its spatial and temporal relevance must then be established, together with whether selective intervention improves clinically meaningful outcomes without compromising repair. Combination therapy should be justified by complementary mechanisms and timing, not by network complexity alone.

## 5. Future Directions

A first priority is to compare permanent occlusion and transient occlusion followed by reperfusion with clearly reported injury duration, sampling region, and outcome window. Interventions initiated at reperfusion or during a defined later interval can help separate acute cardioprotection from altered healing. Serial perfusion assessment, infarct measurements normalized to area at risk, and later functional outcomes address different questions and should not be used interchangeably.

Spatially inferred interactions require further experimental validation to establish a complete communication pathway. In the sender cell, signal generation needs to be disrupted in a cell-specific manner, while signal uptake or receptor function is disrupted in the recipient cell. In addition, extracellular signal transfer should be directly demonstrated, with rescue experiments performed where feasible. For EVs, donor labeling and cargo dependence can be examined at endogenous exposure levels, while metabolite-mediated communication requires isotope tracing and spatial measurements to distinguish intracellular abundance from intercellular transport. Matrix-dependent communication should be tested by linking perturbation of matrix production in a defined source population to recipient mechanosensing and function. Single-cell and spatial RNA, protein, and metabolite measurements should correspond to the same biological intervals and myocardial regions. Cellular adjacency and ligand–receptor coexpression are useful for generating hypotheses, but they cannot determine signal transfer or prove causation. Spatial I/R protein profiling [9], regional metabolomics [19], and MI multiomic maps [7,18] provide complementary information, while differences between experimental models should be taken into account during integration. Clinical translation will require testing selected circuits in animal models representing clinically relevant differences in age, sex, metabolic disease, and treatment exposure, followed by appropriate larger-animal and human studies. A candidate intervention should improve relevant perfusion or injury outcomes without impairing clearance, vascular recovery, or scar integrity. Network complexity alone does not justify combination therapy. Each additional intervention requires evidence of a complementary mechanism and a compatible treatment window.

## 6. Limitations

This is a selective narrative synthesis, and the selection of mechanisms may be influenced by publication and selection biases. Furthermore, the analysis does not provide aggregated estimates of the contributions from various pathways. Extensive literature on spatial distribution characteristics primarily involves permanent coronary artery occlusion or human myocardial infarction (MI) tissue sampled after the very early reperfusion phase. Although these studies characterize the processes of infarction and repair, they cannot isolate reperfusion-induced injury. Even in I/R experiments, measured endpoints may reflect antecedent ischemic injury, reperfusion injury, or subsequent remodeling processes.

The cellular origin and recipient specificity of several mediators remain incompletely defined. Global gene deletion, systemic inhibitors, conditioned media, and administration of purified EVs can affect multiple biological processes and may not establish the physiological contribution of endogenous intercellular communication. Transcript abundance does not confirm receptor activation, and spatial colocalization does not demonstrate functional communication. Animal strain, sex, age, ischemic and reperfusion durations, and comorbidities also vary across studies. Consequently, the relevant schematic diagrams summarize selected mechanisms and reported findings rather than representing quantitatively validated network models or clinical treatment algorithms.

## 7. Conclusions

MIRI reflects the interaction between cell-autonomous injury and spatially and temporally organized intercellular communication. Cell-intrinsic mitochondrial, metabolic, calcium-handling, and death pathways intersect with signaling among cardiomyocytes, immune cells, endothelial cells, platelets, fibroblasts, and vascular mural cells. The consequences of these interactions depend on the identity and state of the sender and recipient cells, the transmitted signal, myocardial location, and temporal phase. A multicellular perspective therefore complements rather than replaces established cell-autonomous mechanisms of reperfusion injury and may help explain why interventions directed at a single pathway or time point often have limited effects.

Clinical translation will require prioritizing intercellular mechanisms with direct causal evidence in reperfused myocardium and defining when and where they can be modified without disrupting necessary inflammatory resolution, vascular repair, scar formation, or functional recovery. Microvascular obstruction already provides a clinically measurable manifestation of persistent tissue-level reperfusion failure with established prognostic relevance after STEMI [44,106], whereas the therapeutic value of selectively targeting border-zone or multicellular signaling remains to be demonstrated. Progress will depend on distinguishing causal communication pathways from spatial association, parallel cellular responses, and cell-autonomous mechanisms, and on testing candidate interventions in models that faithfully reproduce the relevant phase and compartment of human reperfusion injury.

## Data Availability

No new data were created or analyzed in this study. Data sharing is not applicable to this article.

## Abbreviations

MIRI, myocardial ischemia–reperfusion injury; MI, myocardial infarction; PCI, percutaneous coronary intervention; STEMI, ST-segment elevation myocardial infarction; DAMPs, damage-associated molecular patterns; Cx43, connexin 43; ECM, extracellular matrix; GLP-1, glucagon-like peptide-1; NETs, neutrophil extracellular traps; CMR, cardiovascular magnetic resonance; ICAM-1, intercellular adhesion molecule 1; VCAM-1, vascular cell adhesion molecule 1; VWF, von Willebrand factor; EVs, extracellular vesicles; TIMP-1, tissue inhibitor of metalloproteinases-1; CXCR4, C-X-C chemokine receptor 4; RAGE, receptor for advanced glycation end products; sEVs, small extracellular vesicles; cGAS, cyclic GMP-AMP synthase; STING, stimulator of interferon genes; MYD88, myeloid differentiation primary response 88; G-CSF, granulocyte colony-stimulating factor; sgp130Fc, soluble gp130-Fc; MCT4, monocarboxylate transporter 4; MCT1, monocarboxylate transporter 1; ACSL1, acyl-CoA synthetase long-chain family member 1; LPC/LPA, lysophosphatidylcholine/lysophosphatidic acid; Has2, hyaluronan synthase 2; SIRPα, signal regulatory protein α; CypD, cyclophilin D; KATP, ATP-sensitive potassium; HMGB1, high-mobility group box 1; IFN, interferon; mtDNA, mitochondrial DNA; PGC-1α, peroxisome proliferator-activated receptor-γ coactivator 1α; ER, endoplasmic reticulum; CCL2, C-C motif chemokine ligand 2; CD39/ENTPD1, ectonucleoside triphosphate diphosphohydrolase-1; IL-1β, interleukin-1β; TNF, tumor necrosis factor; ADP, adenosine diphosphate; AMP, adenosine monophosphate; ATP, adenosine triphosphate; LV, left ventricular; I/R, ischemia/reperfusion; mPTP, mitochondrial permeability transition pore; ROS, reactive oxygen species; IMH, intramyocardial hemorrhage; MVO, microvascular obstruction.
